# Detection and identification of previously unrecognized microbial pathogens.

**DOI:** 10.3201/eid0403.980310

**Published:** 1998

**Authors:** D A Relman

**Affiliations:** Stanford University, California, USA. relman@cmgm.stanford.edu

## Abstract

Features of a number of important but poorly explained human clinical syndromes strongly indicate a microbial etiology. In these syndromes, the failure of cultivation-dependent microbial detection methods reveals our ignorance of microbial growth requirements. Sequence-based molecular methods, however, offer alternative approaches for microbial identification directly from host specimens found in the setting of unexplained acute illnesses, chronic inflammatory disease, and from anatomic sites that contain commensal microflora. The rapid expansion of genome sequence databases and advances in biotechnology present opportunities and challenges: identification of consensus sequences from which reliable, specific phylogenetic information can be inferred for all taxonomic groups of pathogens, broad-range pathogen identification on the basis of virulence-associated gene families, and use of host gene expression response profiles as specific signatures of microbial infection.


### Detection and Identification of Previously Unrecognized Microbial Pathogens

**David A. Relman**
Stanford University, Stanford, California, USA, and Veterans Affairs Palo Alto Health Care System, Palo Alto, California, USA

Features of a number of important but poorly explained human clinical syndromes strongly indicate a microbial etiology. In these syndromes, the failure of cultivation-dependent microbial detection methods reveals our ignorance of microbial growth requirements. Sequence-based molecular methods, however, offer alternative approaches for microbial identification directly from host specimens found in the setting of unexplained acute illnesses, chronic inflammatory disease, and from anatomic sites that contain commensal microflora. The rapid expansion of genome sequence databases and advances in biotechnology present opportunities and challenges: identification of consensus sequences from which reliable, specific phylogenetic information can be inferred for all taxonomic groups of pathogens, broad-range pathogen identification on the basis of virulence-associated gene families, and use of host gene expression response profiles as specific signatures of microbial infection.

For 100 years, efforts to detect and identify microorganisms have generally begun with the inoculation and incubation of growth media in the laboratory. Colony purification and preparation of limiting dilutions of liquid culture media have provided at least two benefits: amplification of microbial material and purification of single organisms along with their direct descendants. Because some microorganisms are not particular in their growth requirements, these efforts have yielded an array of diverse microbial cultivation types. Serial propagation of microorganisms in the presence of varied energy sources, analysis of their macromolecular composition and their metabolic by-products, and use of specific immunologic reagents have created a variety of systems for microbial classification and identification. Some isolates purified from diseased tissues of animal and human hosts produced identical disease when injected into other, previously healthy hosts. By the latter half of the 20th century, these findings had led to optimism about our ability to detect and recognize microscopic life forms, particularly forms that can cause disease.

Address for correspondence: David A. Relman, VA Palo Alto Health Care System 154T, 3801 Miranda Avenue, Palo Alto, CA 94304, USA; fax: 650-852-3291; e-mail: relman@cmgm.stanford.edu.

Microbial cultivation methods opened up an unsuspected world of microscopic life and presumed causative agents of human illness. However, much of this world remained uncharacterized. In the external environment, certain biochemical activities could best be explained by the presence of microorganisms, although they could not be cultivated in vitro. Sergei Winogradsky, a pioneering soil microbiologist of the early 20th century, spoke about the “less docile” organisms that were not satisfied with laboratory cultivation conditions. In the internal, privileged niches of animals, microorganisms were sometimes visualized in diseased tissues, and persons with typical clinical signs of infection would respond to antibiotics, despite unsuccessful efforts at microbial propagation. That conserved genomic sequences might be used to infer evolutionary ancestry and be amplified directly from natural sites of infection provided the framework for cultivation-independent approaches for microbial detection and identification. In a few years, it became clear that most extant microorganisms in the external environment had been completely overlooked because of their resistance to cultivation on artificial media.

### Sequence-Based Methods for Pathogen Discovery

What features of a genetic sequence make it useful for identifying uncharacterized microorganisms? (1). First, the sequence should be conserved among a relatively large number of known organisms. Second, its rate of change should be constant over long periods and among diverse organisms and should allow inferences of evolutionary distance among a wide range of life forms; the sequence should not be subject to widely discrepant degrees of evolutionary pressure. Third, the sequence should not have been shared among different organisms by horizontal transmission. Finally, the sequence should be amenable to broad-range amplification or detection.

The sequence of the small subunit ribosomal RNA or DNA (ssu rDNA), among other genomic sequences, meets these criteria. Ssu rRNA sequences were the first to reveal a tripartite tree of cellular life, one that includes the bacteria, archaea, and eukarya (2); few genetic sequences reliably reflect the ancestry of such a wide array of cellular life as the ssu rRNA. Since this realization nearly two decades ago, a large ssu rRNA sequence database has accumulated (3), further enhancing the usefulness of this particular locus. (More than 7,000 bacterial 16S rDNA sequences are now available). Highly conserved regions of the ssu rDNA and ssu rRNA provide priming sites for broad-range polymerase chain reaction (PCR) (or RT-PCR) and obviate the need for specific information about a targeted microorganism before this procedure. Thus, a previously uncharacterized bacterium, for example, can be identified from an infected site or tissue by broad range bacterial 16S rDNA amplification, sequencing, and phylogenetic analysis (4). This approach was applied to the uncultivated bacteria of bacillary angiomatosis in 1990 and of Whipple's disease soon thereafter (5,6). Because of the usual presence of host DNA, eukaryotic pathogens (parasites, fungi) must be approached either with domainwide primers and partially purified pathogens or with range (e.g., kingdom)-restricted eukaryotic primers (7).

Broad-range PCR as a method for "pathogen discovery" is not limited to ssu rDNA as a target or to cellular life. Any phylogenetically reliable family of orthologous gene sequences found among a coherent group of microorganisms can be targeted, as long as conserved priming sites can be defined at sites that flank the informative region of sequence. For example, a newly discovered hantavirus was identified as a cause of acute pulmonary disease by using broad-range primers directed at a conserved region of a coat protein-encoding genomic segment (8). A collection of family-restricted broad-range primers is necessary to identify unrecognized viral pathogens; this collection is not yet comprehensive.

Two other independent sequence-based methods are available for pathogen discovery. One relies upon subtractive hybridization to isolate fragments of nucleic acid that are unique (different) to one member of an otherwise matched pair of specimens; these "difference" molecules are then selectively amplified by using linker sequences that had been ligated to all fragments derived from the infected specimen. Multiple rounds of subtraction and amplification are required to find rare fragments within a complex common background. Although better suited than differential display or suppressive subtractive hybridization for low copy targets and highly complex backgrounds (such as human genomic DNA), this method, known as representational difference analysis (RDA) (9), is labor-intensive and cumbersome. Nonetheless, it identified for the first time the presumed causative agent of Kaposi sarcoma, human herpesvirus 8 (9). RDA enables detection of any class of microorganism; however, it may be most useful for DNA viruses. The third sequence-based pathogen discovery method takes advantage of host immunologic recognition of an exogenous microbial agent. Immune sera are used to screen an expression genomic library created from an infected specimen. While laborious, this method has also uncovered an important previously unrecognized pathogen for humans: hepatitis C virus (10).

Sequence-based approaches take advantage of the speed and sensitivity of rapidly evolving molecular biologic methods and the specificity of genotypic characterization. Consensus PCR has the additional advantage of being able to target families of sequences preselected for their reliability in the inference of evolutionary relationships. However, all approaches have limitations. One of the most important for sequence-based methods involves the processing of clinical specimens. Difficulties include heterogeneity of sample, wide variation in the numbers of microbial targets in any given sample, resistance of some microorganisms to digestion and subsequent release of nucleic acid, and presence of PCR inhibitors in varying amounts and types—not to mention ubiquitous microbial nucleic acid contamination of PCR reagents, specimen collection materials, and externally exposed surfaces of the host. These problems reflect the intrinsic biologic variability of a highly complex, partially characterized host. Standardized procedures that produce consistent results with large numbers of clinical specimens are rare. Despite increasing attention to these issues, particularly in the private and commercial sectors, resource commitment and technology advances have lagged behind the development of methods for sequence acquisition and analysis. In fact, it is far easier to generate a putative microbial sequence from a clinical specimen than it is to understand its clinical relevance.

As the process of pathogen discovery and detection turns to the fundamental signature macromolecules of all life forms and away from reliance on cultivation, we increasingly rely on our ability to understand a putative microorganism from its genetic sequence. Many families of virulence-associated genes and gene products are recognizable from their sequence, and their targets are predictable. To predict whether the microorganism whose presence is inferred from amplified genomic fragments is the cause of the disease under study, however, is far more problematic. A replicating organism with which to observe behavior (e.g., drug resistance) and reproduce disease is not available. In fact, the viability of the putative microorganism may not be certain. Although detection of different molecular markers (e.g., specific mRNAs, rRNA/rDNA ratio, resistance-encoding loci) might help resolve some of these questions, it is difficult to determine whether these genotypes and markers all derive from the same organism in that clinical specimen. From a practical standpoint, proof of disease causation from sequence-based investigations will require data that address strength and specificity of association, target dosage effects, temporal considerations, response to therapy, and use of in situ hybridization (11). The selection of proper experimental and control specimens is paramount.

### Settings for Pathogen Discovery

Explorations of microbial diversity within the external environment have yielded surprising results. Nearly all bacteria and archaea revealed by broad-range sequence "mining" in fresh water sites, oceans, surface soils, and deep geologic niches had not been recognized or ever cultivated in the laboratory. Novel kingdoms of life have been discovered with these genotypic methods (12,13). It has been estimated that only 0.4% of all extant bacterial species have been identified. Does this remarkable lack of knowledge pertain to the subset of microorganisms both capable and accomplished in causing human disease? The molecular methods described above could be applied in several settings in which one might expect to find uncharacterized microbial pathogens.

### Acute, Life-Threatening Unexplained Illness

All clinicians are aware of cases characterized by sudden onset of fever, flu-like syndrome, and hemodynamic instability, often accompanied by leukocytosis or leukopenia and rapid deterioration of one or more organ systems. In some cases, despite the strong suggestion of a microbial etiology, conventional diagnostic methods cannot determine the cause. The dramatic nature of these illnesses belies their potential importance to public health and their value in revealing "emerging" agents of disease. An Unexplained Deaths and Critical Illnesses Project has been designed to identify and characterize these illnesses (14). Laboratory investigations include the application of broad-range ssu rDNA PCR. RDA is planned for carefully selected cases with matched control samples. Appropriate specimens have been obtained in only a minority of cases, but positive results from cerebrospinal fluid samples are encouraging. Two lessons have been learned. 1) Well-recognized pathogens may be the cause of some critical illnesses that cannot be explained with traditional diagnostic methods. 2) The process of clinical specimen selection and collection may need to be rethought jointly by molecular biologists and clinicians.

### Chronic Idiopathic Disease

Adaptation and cooptation, features that favor long-term survival of both participants, dominate most host-pathogen relationships. Persistent or intermittent inflammation indicates host perturbation and a subtle imbalance to the relationship and gives rise to clinical manifestations. In fact, the epidemiologic, clinical, and pathologic features of many chronic inflammatory diseases are consistent with a microbial cause, but intimate or symbiotic host-pathogen relationships are among the most difficult to decipher and mimic in the laboratory. Thus, it is not surprising that although microbial etiologies are attractive hypotheses for many chronic diseases, culture-dependent methods have not produced much evidence. Serologic approaches have been useful in providing some leads. For example, the first clues of a possible chlamydial etiology for coronary atherosclerosis were serologic findings. Corroborating data then became available from the use of molecular and in situ methods.

The list of chronic inflammatory diseases with possible microbial etiologies is extensive (15); it includes sarcoidosis, various forms of inflammatory bowel disease, rheumatoid arthritis, systemic lupus erythematosus, Wegener granulomatosis, diabetes mellitus, primary biliary cirrhosis, tropical sprue, and Kawasaki disease. In this discussion, the concept of pathogenic mechanism should be viewed broadly. Many chronic diseases may result from damage or disruption of local immunologic surveillance systems by microbial infection or products; the microorganism is subsequently cleared away, but autoimmune responses or responses directed against commensal flora persist. By the time typical pathologic and clinical findings are produced and the disease is recognized, the inciting agent or its nucleic acids may be gone. Under these circumstances, the optimal time for specimen collection may be well before the disease takes on its characteristic features. Clinical suspicion, astute observation, and identification of disease-predisposing factors are critical. Surprisingly few published studies describe the application of broad-range molecular pathogen discovery methods to the diseases listed above or to other enigmatic chronic disease syndromes. With the finding of microbial sequences in these disease settings, experimental criteria for identifying disease causation must be rigorously pursued (11).

### Commensal Microbial Flora

The human body harbors a 10-fold greater number of microbial cells than human cells. The commensal flora includes microorganisms that occasionally cause disease, especially when host defenses are impaired (due to immunosuppressive drugs, disruption of anatomic barriers, suppression of bacterial flora with antibiotics, or insertion of artificial surfaces). However, in many hosts with impaired conditions and signs and symptoms of infectious disease, an etiologic agent is not identified. If our understanding of microbial diversity within the human-associated commensal flora is as limited as it was of external environments, these clinical observations may not be surprising. That is, the inability to cultivate some of the commensal flora may explain the failure to diagnose related disease. In addition to revolutionizing environmental microbiology, molecular methods may offer rewards for clinical microbiology and the study of internal environmental niches.

Recent research has compared culture-dependent and culture-independent methods of characterizing human commensal flora (16-19). The results suggest that members of at least some phylogenetic groups, e.g., the spirochetes, have been ignored by traditional approaches. Direct comparisons of these two methods will likely show biases and deficiencies with each; nonetheless, important aspects of microbial diversity will be revealed by one and not the other. A complete enumeration of complex microbial communities is not the primary goal. Key members play crucial roles in maintaining the health of the ecosystem (20,21), and understanding community interactions and function may be the more important goal.

### Arthropod Vectors and Small Animal Reservoirs

Several prominent, recently described cultivation-resistant pathogens are transmitted to humans from small animal reservoirs through airborne or vector-borne routes. These pathogens include borreliae (22), bartonellae (23), ehrlichiae, rickettsiae, babesiae, and hantaviruses. These reservoirs and the relevant vectors are attractive targets for pathogen discovery. Searches for restricted groups of microorganisms, searches within restricted host anatomic niches, or searches that include subtractive or differential techniques may be warranted, since all these targets are also hosts for their own commensal (e.g., intestinal) flora. Microorganisms that use arthropod vectors often express different sets of genes within vector versus animal host (e.g., human). Human immune recognition of differentially expressed gene products might help distinguish vector-associated pathogens from nonpathogenic vector-associated flora.

### Phylogenetic Diversity of Microbial Pathogens

Nearly all kingdoms within the domain *Bacteria* contain recognized human pathogens

(Figure). Of those bacterial pathogens identified only by molecular methods, many are clustered within some kingdoms and divisions, such as the alpha-proteobacteria, which include many organisms that form endosymbiotic relationships with their hosts.

Nearly all humans harbor in the intestinal tract Archaea—among the most diverse and numerous cellular life forms on earth (24)—most

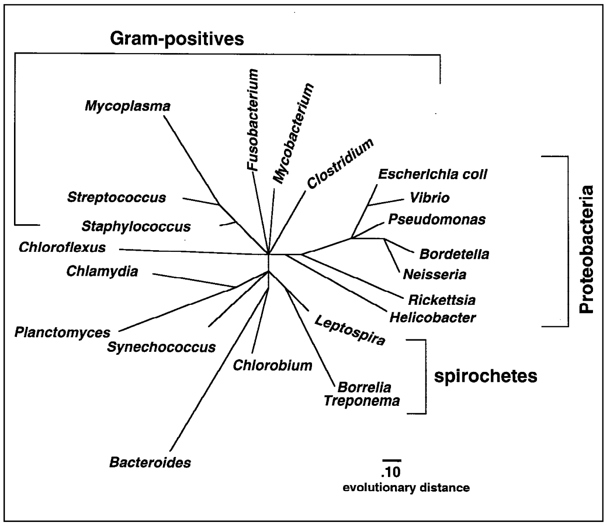

Figure. Evolutionary tree of the domain *Bacteria* based upon comparative analysis of nearly complete 16S rDNA sequences.

notably methanogens. So why are there no known archaeal pathogens? Although some of the most well-known archaea were first identified in (and were assumed to require) extreme environments, they are also found in environments similar to those found within the human body. However, in vitro cultivation methods for many archaea are unavailable, so how would we know if archaeal pathogens existed? Molecular reagents for archaeal detection and identification, i.e., rDNA-based primers and probes, have not been systematically applied to human disease-associated specimens. Without such analyses, finding these organisms in clinical samples would be unlikely.

### Genomics and Newer Technologies

The ultimate genotype of a microorganism is its complete genome sequence. Approximately 15 microbial genomes have been sequenced in their entirety, and the rapid evolution of and large-scale investment in DNA sequencing technology predict full genome sequencing of approximately 50 microorganisms by the year 2000. This massive infusion of primary sequence data unleashes the potential to identify new families of broadly conserved orthologous genes that could be used to infer accurate phylogenies at every level and sector of the evolutionary tree. The number of completed genome sequences is too small to effect this goal (25). The sequence data sets for newly characterized genes are too small to assess the reliability of the phylogenies they predict. The problem imposed by horizontal gene transfer is now more apparent with the analysis of multitudes of gene families. To identify a well-characterized microorganism, an exact genotypic "hit" with a highly variable locus is sufficient. Likewise, clonality and clone identification can be determined with sequences from collections of polymorphic, but conserved loci, e.g., "housekeeping genes" (26). But for an unrecognized organism, the sequence locus or loci selected for genotyping must be highly conserved and phylogenetically informative and reliable. Over the next 5 years, with the increasing use of large-scale comparative genomic techniques, microbial sequence databases will represent the broad diversity among distant ancestral relatives, as well as the fine differences among closely related cousins. Assessment of putative universal sequences can be undertaken. All these developments and future trends apply equally well to the wide array of animal viruses and viral genomes (Table 1). As genotypes become more easily interpreted, they will continue to displace phenotypic characterization as the basis for pathogen recognition.

Often the only difference between a pathogenic and a nonpathogenic strain of the same species, e.g., enteropathogenic and nonenteropathogenic *Escherichia coli*, is a small set of virulence genes. These differences are not reflected in the ancestry inferred from more stable chromosomal markers (Table 2). Yet detection of these genes is a fundamental aspect

Table 1. Newer diagnostic technologies

| |
|---|
| 1. High-density DNA microarrays |
| • broad-based pathogen detection and characterization: bacteria, eukarya, viruses |
| • virulence-associated gene families |
| • comprehensive host gene expression profiles |
| 2. Improved nucleic acid subtractive methods |
| 3. Novel bioassays for toxin activity |
| • neurons– or myocytes–on-a-chip |

of pathogen identification. Microbial virulence is a phenotype whose genetic basis is rapidly being revealed. Families of virulence-associated genes responsible for microbial adherence, toxicity, specialized secretion, environmental sensing, and subversion of immune defenses have been defined, albeit with many sequence variations on a theme (27,28). One of the most important features of these genes is their proclivity toward horizontal transfer and over relatively rapid time scales. Genome sequencing efforts have facilitated, and will continue to facilitate, this approach to pathogen discovery. Physical clusters (or islands) of virulence genes are being identified, and their distinctive composition and boundaries are being defined (29). One might well imagine the development of a comprehensive set of consensus primers and probes for detecting these gene families, clusters, and islands (Table 1).

With increasing value placed on genotypic information and increasing numbers of potentially useful genotyping loci, the technology of sequence determination and primary genomic characterization has assumed center stage. Goals include speed, convenience, and large-scale sequencing. High density DNA microarray technology is one of the most promising in this context (Table 1). Depending on the format, microarrays can be used to detect nucleic acid polymorphisms or to sequence de novo; they can also quantitate mRNA. At least two basic applications of DNA microarray technology are available for pathogen detection and identification; neither has been fully developed or tested clinically (Table 1). The first would consist of a set of probes designed to assess ssu rDNA sequence diversity of all known monophyletic groups of bacteria, archaea, viruses, and nonanimal eukarya. Other phylogenetically reliable loci might be substituted for rDNA or included as well. In addition, consensus probes for families of virulence-associated genes, as described above, would facilitate identification of unsuspected or newly acquired pathogenic attributes in organisms not usually associated with these traits. Differential hybridizations and multiple fluorophores allow easy detection of hybridized target and normalization of quantified values to a reference sample. This sort of broad-range "pathogen detection chip" would identify mixed infections, as well as chimeric or novel microorganisms (Table 2); it could rapidly create an inventory of highly complex microbial communities and measure changes in individual members as a function of varying environmental conditions.

Table 2. Pathogens that may be difficult to detect or identify

1. Pathogens that establish intimate relationships with the host
    - endosymbionts and intracellular organisms
2. Chimeras: natural versus man-made?
3. "Nonpathogens" that acquire virulence-associated genes
4. Microorganisms without "universal" sequences?

The second theoretical use of DNA microarray technology for pathogen detection would focus on host gene responses. Arrays in current use at academic and commercial research laboratories are capable of quantitating expression responses by 10,000 to 20,000 human genes simultaneously (30-34). During most infectious diseases, directly affected tissues, secondary sites, and circulating leukocytes will likely display sets of common nonspecific expression responses; however, since each microbial pathogen interacts with and manipulates the host in a complex and unique manner, within these highly complex patterns there will also likely be critical diagnostic signatures that distinguish infection by one pathogen from infection by another. Furthermore, these stereotypic expression patterns will evolve. The time of initial host exposure to a pathogen might be determined by comparing new expression patterns with a suitable preexisting set of timed profiles. Patterns will provide clues about the pathogenesis of chronic inflammatory disease (35). Through the identification of key response genes might emerge novel diagnostic assays for their putative protein products and novel strategies for interfering with or blocking disease pathogenesis.

In many cases, infection-associated tissue damage occurs in the absence of intact microorganisms. Toxin-mediated disease is a prominent example. Often, microbial toxins act at a distance from the original site of microbial toxin production and release. In this setting, genotypic approaches for microbial detection may not be appropriate; in addition to the assessment of host responses, novel bioassays for toxin activity are attractive options (Table 1). For example, in a system designed by Greg Kovacs at Stanford University, neurons or myocytes are cultivated on the electrical contacts of a miniaturized circuit board. The electrical output and properties of these cells can be monitored and analyzed as they are exposed to diverse membrane-active toxins. Although this technology is at an early stage of development, we know that such cells are extremely sensitive to chemical toxins, and this sensitivity can be recorded in the form of altered action potentials and changes in impedance and cell movement. Experiments are under way to test cell responses to biologic toxins in a variety of clinically relevant experimental conditions.

### Relationships between Pathogen and Host

As more sensitive and comprehensive methods for uncovering human-associated pathogenic microorganisms identify previously unsuspected host-pathogen relationships, the nature of these relationships may need to be rethought (36,37). Parasitism and commensalism are probably not the complete story; mutualism may be more common in the human host than is usually taught. Evidence of coevolution between host and microbe suggests codependence. The endosymbiont theory for the origin of eukaryotic organelles is consistent with the same (38). Microbial remnants and cryptic genomic fragments may not be so uncommon within the human genome; for example, approximately 1% of the human genome is retrovirus sequence (39). Some of these viral genes may be expressed during local inflammation. The real challenges in pathogen discovery will be the problems of sequence interpretation, clinical relevance, and proof of causation. In the end, pathogen discovery will by necessity be a multidisciplinary effort (40). Only with the coordinated interaction of epidemiologists, pathologists, and clinicians will the role of microorganisms in disease be clearly defined.

David A. Relman is assistant professor of medicine and of microbiology and immunology at Stanford University, Stanford, California; he is also staff physician at Veterans Affairs Palo Alto Health Care System, Palo Alto, California. His research interests include Bordetella pathogenesis, molecular and genomic aspects of pathogen recognition by the host, and development and application of molecular methods for the identification of unrecognized microbial pathogens.

### References

1. Woese CR. Bacterial evolution. Microbiol Rev 1987;51:221-71.
2. Woese CR, Kandler O, Wheelis ML. Towards a natural system of organisms: proposal for the domains Archaea, Bacteria, and Eucarya. Proc Natl Acad Sci U S A 1990;87:4576-9.
3. Maidak BL, Olsen GJ, Larsen N, Overbeek R, McCaughey MJ, Woese CR. The RDP (Ribosomal Database Project). Nucleic Acids Res 1997;25:109-11.
4. Relman DA, Loutit JS, Schmidt TM, Falkow S, Tompkins LS. The agent of bacillary angiomatosis. An approach to the identification of uncultured pathogens. N Engl J Med 1990;323:1573-80.
5. Relman DA, Schmidt TM, MacDermott RP, Falkow S. Identification of the uncultured bacillus of Whipple's disease. N Engl J Med 1992;327:293-301.
6. Wilson KH, Blitchington R, Frothingham R, Wilson JA. Phylogeny of the Whipple's-disease-associated bacterium. Lancet 1991;338:474-5.
7. Santamaria-Fries M, Fajardo LF, Sogin ML, Olson PD, Relman DA. Lethal infection by a previously unrecognised metazoan parasite. Lancet 1996;347:1797-801.
8. Nichol ST, Spiropoulou CF, Morzunov S, Rollin PE, Ksiazek TG, Feldmann H, et al. Genetic identification of a hantavirus associated with an outbreak of acute respiratory illness. Science 1993;262:914-7.
9. Chang Y, Cesarman E, Pessin MS, Lee F, Culpepper J, Knowles DM, et al. Identification of herpesvirus-like DNA sequences in AIDS-associated Kaposi's sarcoma. Science 1994;266:1865-9.
10. Choo QL, Kuo G, Weiner AJ, Overby LR, Bradley DW, Houghton M. Isolation of a cDNA clone derived from a blood-borne non-A, non-B viral hepatitis genome. Science 1989;244:359-62.
11. Fredricks DN, Relman DA. Sequence-based identification of microbial pathogens: a reconsideration of Koch's postulates. Clin Microbiol Rev 1996;9:18-33.
12. Barns SM, Delwiche CF, Palmer JD, Pace NR. Perspectives on archaeal diversity, thermophily and monophyly from environmental rRNA sequences. Proc Natl Acad Sci U S A 1996;93:9188-93.
13. Hugenholtz P, Pitulle C, Hershberger KL, Pace NR. Novel division level bacterial diversity in a Yellowstone hot spring. J Bacteriol 1998;180:366-76.
14. Perkins BA, Flood JM, Danila R, Holman RC, Reingold AL, Klug LA, et al. Unexplained deaths due to possibly infectious causes in the United States: defining the problem and designing surveillance and laboratory approaches. The Unexplained Deaths Working Group. Emerg Infect Dis 1996;2:47-53.
15. Fredricks DN, Relman DA. Infectious agents and the etiology of chronic idiopathic diseases. In: Remington JS, Swartz MN, editors. Current Clinical Topics in Infectious Diseases. Vol. 18. Boston: Blackwell Scientific Publications. In press 1998.
16. Choi BK, Paster BJ, Dewhirst FE, Gobel UB. Diversity of cultivable and uncultivable oral spirochetes from a patient with severe destructive periodontitis. Infect Immun 1994;62:1889-95.
17. Hugenholtz P, Pace NR. Identifying microbial diversity in the natural environment: a molecular phylogenetic approach. Trends in Biotechnology 1996;14:190-7.

18. Millar MR, Linton CJ, Cade A, Glancy D, Hall M, Jalal H. Application of 16S rRNA gene PCR to study bowel flora of preterm infants with and without necrotizing enterocolitis. J Clin Microbiol 1996;34:2506-10.
19. Wilson KH, Blitchington RB. Human colonic biota studied by ribosomal DNA sequence analysis. Appl Environ Microbiol 1996;62:2273-8.
20. Tilman D, Knops J, Wedin D, Reich P, Ritchie M, Siemann E. The influence of functional diversity and composition on ecosystem processes. Science 1997;277:1300-2.
21. Wardle DA, Zackrisson O, Hörnberg G, Gallet C. The influence of island area on ecosystem properties. Science 1997;277:1296-9.
22. Barbour AG, Maupin GO, Teltow GJ, Carter CJ, Piesman J. Identification of an uncultivable Borrelia species in the hard tick *Amblyomma americanum*: possible agent of a Lyme disease-like illness. J Infect Dis 1996;173:403-9.
23. Hofmeister EK, Kolbert CP, Abdulkarim AS, Magera JM, Hopkins MK, Uhl JR, et al. Cosegregation of a novel *Bartonella* species with *Borrelia burgdorferi* and *Babesia microti* in *Peromyscus leucopus*. J Infect Dis 1998;177:409-16.
24. Olsen GJ, Woese CR. Archaeal genomics: an overview. Cell 1997;89:991-4.
25. Tatusov RL, Koonin EV, Lipman DJ. A genomic perspective on protein families. Science 1997;278:631-7.
26. Maiden MC, Bygraves JA, Feil E, Morelli G, Russell JE, Urwin R, et al. Multilocus sequence typing: a portable approach to the identification of clones within populations of pathogenic microorganisms. Proc Natl Acad Sci U S A 1998;95:3140-5.
27. Finlay BB, Falkow S. Common themes in microbial pathogenicity revisited. Microbiol Mol Biol Rev 1997;61:136-69.
28. Relman DA, Falkow S. A molecular perspective of microbial pathogenicity. In: Mandell GL, Douglas RG, Bennett JE, editors. Principles and practice of infectious diseases. 4th ed. New York: Churchill Livingstone; 1994. p. 19-29.
29. Groisman EA, Ochman H. Pathogenicity islands: bacterial evolution in quantum leaps. Cell 1996;87:791-4.
30. Chee M, Yang R, Hubbell E, Berno A, Huang XC, Stern D, et al. Accessing genetic information with high-density DNA arrays. Science 1996;274:610-4.
31. DeRisi J, Penland L, Brown PO, Bittner ML, Meltzer PS, Ray M, et al. Use of a cDNA microarray to analyse gene expression patterns in human cancer. Nat Genet 1996;14:457-60.
32. Schena M, Shalon D, Davis RW, Brown PO. Quantitative monitoring of gene expression patterns with a complementary DNA microarray. Science 1995;270:467-70.
33. Schena M, Shalon D, Heller R, Chai A, Brown PO, Davis RW. Parallel human genome analysis: microarray-based expression monitoring of 1000 genes. Proc Natl Acad Sci U S A 1996;93:10614-9.
34. Shalon D, Smith SJ, Brown PO. A DNA microarray system for analyzing complex DNA samples using two-color fluorescent probe hybridization. Genome Res 1996;6:639-45.
35. Heller RA, Schena M, Chai A, Shalon D, Bedilion T, Gilmore J, et al. Discovery and analysis of inflammatory disease-related genes using cDNA microarrays. Proc Natl Acad Sci U S A 1997;94:2150-5.
36. Krause RM. Dynamics of emergence. J Infect Dis 1994;170:265-71.
37. Lederberg J. Infectious disease as an evolutionary paradigm. Emerg Infect Dis 1997;3:417-23.
38. Gray MW. The endosymbiont hypothesis revisited. Int Rev Cytol 1992;141:233-357.
39. Lower R, Lower J, Kurth R. The viruses in all of us: characteristics and biological significance of human endogenous retrovirus sequences. Proc Natl Acad Sci U S A 1996;93:5177-84.
40. Fredricks DN, Relman DA. Infectious agents and the etiology of chronic idiopathic diseases. Curr Clin Top Infect Dis 1998;18:180-200.

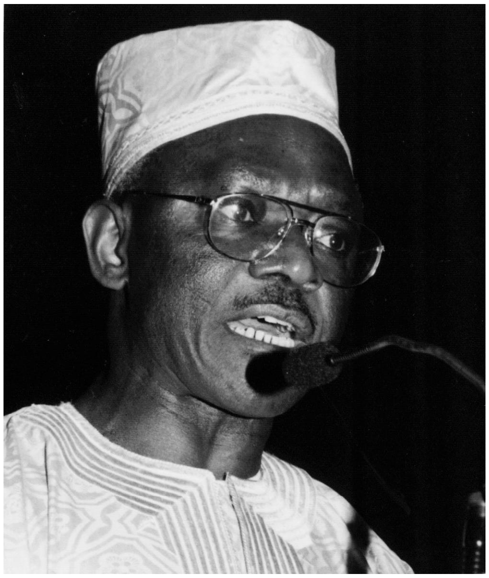

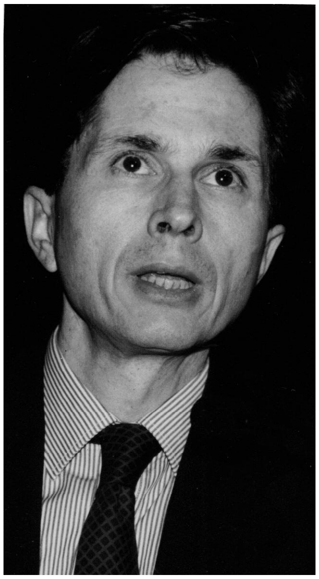

Thomas C. Nchinda (L)
David Heymann
World Health Organization,
Geneva, Switzerland.

## References

[PDF_00710] Barbour A. G., Maupin G. O., Teltow G. J., Carter C. J., Piesman J. (1996). Identification of an uncultivable Borrelia species in the hard tick Amblyomma americanum: possible agent of a Lyme disease-like illness.. J Infect Dis.

[PDF_00670] Barns S. M., Delwiche C. F., Palmer J. D., Pace N. R. (1996). Perspectives on archaeal diversity, thermophily and monophyly from environmental rRNA sequences.. Proc Natl Acad Sci U S A.

[PDF_00659] Chang Y., Cesarman E., Pessin M. S., Lee F., Culpepper J., Knowles D. M., Moore P. S. (1994). Identification of herpesvirus-like DNA sequences in AIDS-associated Kaposi's sarcoma.. Science.

[PDF_00739] Chee M., Yang R., Hubbell E., Berno A., Huang X. C., Stern D., Winkler J., Lockhart D. J., Morris M. S., Fodor S. P. (1996). Accessing genetic information with high-density DNA arrays.. Science.

[PDF_00688] Choi B. K., Paster B. J., Dewhirst F. E., Göbel U. B. (1994). Diversity of cultivable and uncultivable oral spirochetes from a patient with severe destructive periodontitis.. Infect Immun.

[PDF_00663] Choo Q. L., Kuo G., Weiner A. J., Overby L. R., Bradley D. W., Houghton M. (1989). Isolation of a cDNA clone derived from a blood-borne non-A, non-B viral hepatitis genome.. Science.

[PDF_00742] DeRisi J., Penland L., Brown P. O., Bittner M. L., Meltzer P. S., Ray M., Chen Y., Su Y. A., Trent J. M. (1996). Use of a cDNA microarray to analyse gene expression patterns in human cancer.. Nat Genet.

[PDF_00729] Finlay B. B., Falkow S. (1997). Common themes in microbial pathogenicity revisited.. Microbiol Mol Biol Rev.

[PDF_00772] Fredricks D. N., Relman D. A. (1998). Infectious agents and the etiology of chronic idiopathic diseases.. Curr Clin Top Infect Dis.

[PDF_00667] Fredricks D. N., Relman D. A. (1996). Sequence-based identification of microbial pathogens: a reconsideration of Koch's postulates.. Clin Microbiol Rev.

[PDF_00766] Gray M. W. (1992). The endosymbiont hypothesis revisited.. Int Rev Cytol.

[PDF_00737] Groisman E. A., Ochman H. (1996). Pathogenicity islands: bacterial evolution in quantum leaps.. Cell.

[PDF_00758] Heller R. A., Schena M., Chai A., Shalon D., Bedilion T., Gilmore J., Woolley D. E., Davis R. W. (1997). Discovery and analysis of inflammatory disease-related genes using cDNA microarrays.. Proc Natl Acad Sci U S A.

[PDF_00715] Hofmeister E. K., Kolbert C. P., Abdulkarim A. S., Magera J. M., Hopkins M. K., Uhl J. R., Ambyaye A., Telford S. R., Cockerill F. R., Persing D. H. (1998). Cosegregation of a novel Bartonella species with Borrelia burgdorferi and Babesia microti in Peromyscus leucopus.. J Infect Dis.

[PDF_00692] Hugenholtz P., Pace N. R. (1996). Identifying microbial diversity in the natural environment: a molecular phylogenetic approach.. Trends Biotechnol.

[PDF_00674] Hugenholtz P., Pitulle C., Hershberger K. L., Pace N. R. (1998). Novel division level bacterial diversity in a Yellowstone hot spring.. J Bacteriol.

[PDF_00762] Krause R. M. (1994). Dynamics of emergence.. J Infect Dis.

[PDF_00764] Lederberg J. (1997). Infectious disease as an evolutionary paradigm.. Emerg Infect Dis.

[PDF_00768] Löwer R., Löwer J., Kurth R. (1996). The viruses in all of us: characteristics and biological significance of human endogenous retrovirus sequences.. Proc Natl Acad Sci U S A.

[PDF_00639] Maidak B. L., Olsen G. J., Larsen N., Overbeek R., McCaughey M. J., Woese C. R. (1997). The RDP (Ribosomal Database Project).. Nucleic Acids Res.

[PDF_00724] Maiden M. C., Bygraves J. A., Feil E., Morelli G., Russell J. E., Urwin R., Zhang Q., Zhou J., Zurth K., Caugant D. A. (1998). Multilocus sequence typing: a portable approach to the identification of clones within populations of pathogenic microorganisms.. Proc Natl Acad Sci U S A.

[PDF_00697] Millar M. R., Linton C. J., Cade A., Glancy D., Hall M., Jalal H. (1996). Application of 16S rRNA gene PCR to study bowel flora of preterm infants with and without necrotizing enterocolitis.. J Clin Microbiol.

[PDF_00655] Nichol S. T., Spiropoulou C. F., Morzunov S., Rollin P. E., Ksiazek T. G., Feldmann H., Sanchez A., Childs J., Zaki S., Peters C. J. (1993). Genetic identification of a hantavirus associated with an outbreak of acute respiratory illness.. Science.

[PDF_00720] Olsen G. J., Woese C. R. (1997). Archaeal genomics: an overview.. Cell.

[PDF_00677] Perkins B. A., Flood J. M., Danila R., Holman R. C., Reingold A. L., Klug L. A., Virata M., Cieslak P. R., Zaki S. R., Pinner R. W. (1996). Unexplained deaths due to possibly infectious causes in the United States: defining the problem and designing surveillance and laboratory approaches. The Unexplained Deaths Working Group.. Emerg Infect Dis.

[PDF_00642] Relman D. A., Loutit J. S., Schmidt T. M., Falkow S., Tompkins L. S. (1990). The agent of bacillary angiomatosis. An approach to the identification of uncultured pathogens.. N Engl J Med.

[PDF_00646] Relman D. A., Schmidt T. M., MacDermott R. P., Falkow S. (1992). Identification of the uncultured bacillus of Whipple's disease.. N Engl J Med.

[PDF_00652] Santamaría-Fríes M., Fajardo L. F., Sogin M. L., Olson P. D., Relman D. A. (1996). Lethal infection by a previously unrecognised metazoan parasite.. Lancet.

[PDF_00746] Schena M., Shalon D., Davis R. W., Brown P. O. (1995). Quantitative monitoring of gene expression patterns with a complementary DNA microarray.. Science.

[PDF_00750] Schena M., Shalon D., Heller R., Chai A., Brown P. O., Davis R. W. (1996). Parallel human genome analysis: microarray-based expression monitoring of 1000 genes.. Proc Natl Acad Sci U S A.

[PDF_00754] Shalon D., Smith S. J., Brown P. O. (1996). A DNA microarray system for analyzing complex DNA samples using two-color fluorescent probe hybridization.. Genome Res.

[PDF_00722] Tatusov R. L., Koonin E. V., Lipman D. J. (1997). A genomic perspective on protein families.. Science.

[PDF_00701] Wilson K. H., Blitchington R. B. (1996). Human colonic biota studied by ribosomal DNA sequence analysis.. Appl Environ Microbiol.

[PDF_00649] Wilson K. H., Blitchington R., Frothingham R., Wilson J. A. (1991). Phylogeny of the Whipple's-disease-associated bacterium.. Lancet.

[PDF_00633] Woese C. R. (1987). Bacterial evolution.. Microbiol Rev.

[PDF_00635] Woese C. R., Kandler O., Wheelis M. L. (1990). Towards a natural system of organisms: proposal for the domains Archaea, Bacteria, and Eucarya.. Proc Natl Acad Sci U S A.

